# Exploring Maternal and Child Health Among Tribal Communities in India: A Life Course Perspective

**DOI:** 10.5539/gjhs.v16n2p31

**Published:** 2024-01-04

**Authors:** Minal Madankar, Narendra Kakade, Lohitha Basa, Bushra Sabri

**Affiliations:** 1 Tata Institute of Social Sciences, Mumbai, India; 2 School of Nursing, Johns Hopkins University, Baltimore, Maryland, USA

**Keywords:** child health, healthcare access, maternal health, pregnancy, tribal communities, tribal health

## Abstract

India experiences high rates of maternal and infant mortality and morbidity, with tribal communities disproportionately affected. Tribal populations frequently live in unfavorable socio-economic conditions and deficient social health indicators, culminating in adverse health consequences. Using a life course perspective, this qualitative study explored risks over the life course that contribute to maternal and child health problems among tribal populations in India. Additionally, the study examined barriers to utilization of healthcare services during the pregnancy and postpartum periods. Data collection occurred between 2017 and 2019 through participant observation, key informant interviews (*n* = 7) and in-depth interviews (*n* = 68) and a focus group (*n* = 7) with tribal women from the Madia-Gond tribe in the Indian state of Maharashtra. Additionally, verbal autopsies were conducted with relatives of three deceased women and five infants from the tribe. Multiple risk factors operating at different socio-ecological levels and developmental stages of life were associated with maternal and child health problems among the tribe. These included adherence to traditional harmful practices, limited access to nutritional diet, women’s health neglected due to the double burden of domestic and professional labor, and a lack of accessible and well-equipped medical facilities. Inaccesibility stemmed from factors including extreme poverty, geographical isolation, and suboptimal healthcare infrastructure. There is need for provisions to promote access to care and to promote education and awareness centered on evidence-supported healthcare, particularly targeted towards expectant mothers. The implementation of nutritional support programs may help mitigate high maternal and child mortality and morbidity rates prevalent among tribal populations.

## 1. Introduction

Maternal and infant mortalities pose substantial challenges to global public health. The world suffers from an unexpectedly high burden of maternal mortality, with an overall estimated maternal mortality ratio (MMR) of 223 maternal deaths per 100 000 live births in the year 2020 (World Health Organization [WHO], 2023). Central and Southern Asia are one of the biggest contributors to global maternal mortality with an MMR of approximately 129 (WHO, 2023). In 2020, India recorded the second-highest number of global maternal deaths, with approximately 24,000 fatalities, ranking just below Nigeria (WHO, 2023). Similarly, India also has one of the highest global infant mortality ratios (IMR) standing at 40.7 deaths per 1000 live births (Ministry of Tribal Affairs, 2023). Tribal communities in India bear a significant burden, as they account for over 50% of all maternal deaths and IMR in the country (Dasra, 2016). The IMR among tribal children is 30% higher than the national average and 61% higher for tribal children under five (Dasra, 2016). The higher IMR and MMR observed in tribal populations can be attributed to disparities in social determinants of health such as education, adequate and trained healthcare workforce, access to care, and health financing (Dasra, 2016; Kumar et al., 2020). Inadequate healthcare infrastructure, limited access to essential services, malnutrition, and high disease prevalence, hinder overall health and well-being of tribal populations in India (Hamal et al., 2020). To illustrate, maternal and child healthcare services are mostly underutilized amongst tribal women, with only 10 % of tribal women receiving full antenatal care and only 18% of tribal women having institutional deliveries (Dasra, 2016). Tribal children were found to have a full vaccination rate of only 55.8%, whereas the national average stood at 62.0% (Ministry of Tribal Affairs, 2019). The elevated IMR in tribal communities can be partially attributed to the lower vaccination rates.

The presence of unfavorable social indicators in tribal populations is the result of long-standing discrimination and an infringement of rights of tribal communities. Tribal populations endure the most of systemic and structural marginalization within Indian society due to land disputes, socio-economic disparities, and cultural differences (Søreide, 2017). Social and geographic isolation adversely affects maternal and infant health in tribal communities (Hamal et al., 2020). Further, almost 90% of tribal people reside in rural areas (The Expert Committee on Tribal Health, 2023). Rural areas in India are characterized by numerous healthcare challenges, including limited access to quality healthcare facilities, subpar quality of primary health care, and ineffective training of rural healthcare professionals (Mohan & Kumar, 2019; Sabri et al., 2023). The intersectionality of belonging to a tribe, with low socioeconomic status, and living in a forest or a remote rural area compounds the disease burden, result in a quadruple challenge for maternal and child health care among tribal populations. This includes communicable and non-communicable diseases, malnutrition, mental health issues, and addictions, all exacerbated by inadequate health-seeking behaviors and healthcare infrastructure (Kumar et al., 2020).

The life course perspective is a useful framework for examining multiple factors throughout the life span that contribute to disparities in maternal and child health outcomes among the tribes. Current literature lacks comprehensive coverage and analysis of factors related maternal and child health in tribal populations in India. Moreover, there is a lack of qualitative literature that provides first-hand accounts and detailed descriptions of the hardships and challenges experienced by tribal women and children. This study offers valuable insights to practitioners regarding the specific gaps faced by rural, tribal women, and their children when it comes to accessing necessary healthcare services. By enhancing policy makers and practitioners’ understanding of the conditions tribal people face, the findings from the study informs the development of essential infrastructure and initiatives aimed at reducing health disparities among tribal women and children. Therefore, the purpose of this qualitative study was to identify risks over the life course that contribute to maternal and child mortalities and morbidities from the perspective of primary healthcare workers, and women and families residing in tribal areas in India. Using a life course approach (Jones et al., 2019), this study collected data to gain insight into the socio-demographic, economic, cultural, and behavioral factors interwoven throughout women’s life cycles. These factors play a crucial role in shaping maternal and child health outcomes and, in some instances, can lead to expected or unexpected tragic events, such as death.

## 2. Method

### 2.1 Study Design, Setting, and Sample

This qualitative study, part of a mixed methods project (2017–2019), aimed to understand poor maternal and child health (MCH) among tribal women and children in Bhamaragadh, one of the most socio-economically underdeveloped areas in the Indian state of Maharashtra. Bhamaragadh, home to the Madia-Gond tribes, faces isolation due to political insurgency and police conflict (Soreide, 2013). The region’s challenges are compounded by dense forest coverage, limited mobile telecommunicatiaon connectivity, inadequate transportation, and regular power outages (Government of Maharashtra, 2023). As evidenced by poor MCH indicators including high maternal and neonatal mortality rates, the geographical location and limited resource accessibility, pose health challenges for Madia-Gond families (Kalkonde et al., 2019). This study used an integrated research paradigm for understanding women’s lived experiences in Bhamaragarh. The process involved a phenomenological and narrative design with an interpretivist approach to explore MCH issues in the area. Data collection involved key informant interviews (*n* = 7), verbal autopsy interviews (*n* = 7), focus group (*n* = 1, with 7 participants), in-depth interviews (*n* = 68) and participant observation. Participants were selected using purposive sampling, with the sample including seven primary healthcare workers from the key informant interviews, eight family members of three deceased women and five infants from the verbal autopsy interviews, seven tribal women from a focus group and 68 tribal women participants from the in-depth interviews. The key informants were eligible if they provided primary healthcare services to Madia-Gond tribal families in the study area. These included Auxiliary Nurse Midwives (ANMs; *n* = 3), General Nursing and Midwife (GNM; *n* = 1), Accredited Social Healthcare Activists (ASHAs; *n* = 1) and Multipurpose Workers (MPW, *n* = 2). The verbal autopsy interviews were conducted with immediate family members of deceased women or children from the Madia-Gond tribe. Only those families in which death of a mother or a child occured within the past year were selected for the interviews. The eligibility criteria for the focus group were (1) women from the Madia-Gond tribe and (2) recent (past year) delivery or currently pregnant. The eligibility criteria for women’s in-depth interviews were (1) women from the Madia-Gond tribe and (2) have delivered a child within the past year.

### 2.2 Recruitment and Data Collection Procedure

For recruitment, the researcher (a trained Master of Public Health student) met with the Block Health officer of Bhamaragadh, who called a meeting of 19 health care workers from the three primary healthcare centers covering 24 tribal villages. The meeting with the workers involved sharing information about the study and getting their buy-in for assistance with identifying and consenting potential study participants. The researcher also stayed with the community for 80 days (April to June, 2019). During this stay, she closely observed the day-to -day activities of Madia-Gond women, including their access to MCH services in the community and maintained a diary. She contacted the participants at least a day in advance with the help of village health workers. On average, 3–4 interviews were conducted per day with participants from 1–3 villages, depending upon the proximity of the villages and number of participants available for the interview. Data collection in unapproachable or hard to reach villages involved a week-long stay in those villages for recruitment.

After obtaining oral consent, data was collected in-person at a private location using semi-structured interview and focus group guides, with sessions lasting for approximately 60–90 minutes. The interviews and focus group were conducted in Gondi and Marathi language by the researcher. The primary healthcare worker who was trusted by women served as the interpreter for Gondi, the local tribal language. Drawing from the life course perspective (Jones et al., 2019), the questions in interviews and focus groups explored the factors or experiences and trajectory that shaped MCH outcomes for tribal populations. All the interviews examined factors on a continuum from mother’s womb to birth - childhood - adolescence - married life - conception - delivery and in some cases to unfortunate deaths of woman. The key informant interviews elicited providers’ perspectives in delivering healthcare services in the tribal region and their experiences and views about the social, behavioral, economic, cultural, and physical health conditions of women. The in-depth interviews and focus group for women explored socioeconomic, cultural, and structural barriers to MCH in the tribe, and access to and utilization of antenatal, intra-natal and postnatal care. The sessions also explored strategic approaches to prenatal and post-natal care of women and children in the tribal region. The sessions were audio-recorded, translated, and transcribed verbatim in English. All study procedures were approved by the Tata Institute of Social Science Faculty Ethics Committee.

### 2.3 Data Analysis Procedures

Data was analyzed using content analysis procedure (Bengtsson, 2016). The focus was on understanding the impact of multilevel factors such as socio-economic conditions, cultural practices, and prenatal to postnatal care on MCH in Bhamaragadh’s tribal families. Using the life course perspective (Jones et al., 2019), the analysis identified multiple factors that influenced and affected Madia-Gond women such as being in their mother’s womb to their birth - childhood - adolescence - early marriage - early pregnancy and multiple deliveries in underage period - delivery complications and sometimes to deaths. The researcher did not create a codebook a priori and used a data-driven approach to discover patterns and themes in participants’ responses to the questions and follow up questions. Themes were systematically aligned with segments of the life-course and socio-ecological models. Triangulation from three qualitative sources (in-depth interviews, focus group, verbal autopsies) ensured the trustworthiness of the findings.

## 3. Results

### 3.1 The Childhood Experience of Madia-Gond Girls

The risks experienced by Madia-Gond women starts from birth itself, with preterm births and low-birth weight babies during delivery being a prevalent phenomenon. Bhamaragadh’s neonatal mortality is high, accounting for 12.4% of Maharashtra’s total neonatal deaths (Saunik et al., 2017).

#### 3.1.1 Socio-cultural Level

##### Impact of Traditional Childcare Practices:

Traditional tribal cultural practices negatively impact the health outcomes of infants. For example, tribal women often bathe newborns believing they are covered in harmful fluids and dirt, risking infection and hypothermia for the baby (Priyadarshi et al., 2022). Another issue is the practice of delayed breastfeeding. Table 1 (quote 1) indicates that women often discard colostrum- the initial thick, sticky, and pale, yellow breast milk- seen as indigestible waste milk, despite its rich nutrients and immune-boosting properties for newborns (Cleveland Clinic, 2022). Tribal women do not breastfeed. Instead, they provide pre-lacteal feeds such as honey, sugar water mixed with traditional herbs, animal milk, and even formula as seen in Table 1 (quote 2). Breastfeeding is further complicated by the high rate of anemia in mothers, with 65% of all tribal women aged 15–49 having anemia, resulting in insufficient breast milk production (The Expert Committee on Tribal Health, 2023). There is a notable lack of breastfeeding among tribal women, which significantly compromises their nutrition and contributes to poor survival rates for newborn.

#### 3.1.3 Community Level

##### Limited Access to Nutritional Diet and Healthcare Services:

Due to poverty and resource scarcity, Madia-Gond children often face malnourishment from insufficient nutritious food. Only 4.5% of Madia-Gond children aged 6–23 months receive an adequate diet, manifesting in 35.4% of children being underweight (National Family Healthy Survey-5 (NFHS-5), 2020). The lack of nutritious food also causes developmental delays and impairments in cognitive functions, with the under-5 stunting rate in the rural Gadchiroli district being 35.7% (NFHS-5, 2020). Proper nutrition and early childhood development activities are crucial for a child’s well-being and health. However, postnatal services and nutritional support from the public health department struggle to reach tribal children due to insurgency and challenging terrain. Table 1 (quotes 3 and 4) depicts the lack of nutritious food available for Madia-Gond children.

##### Limited Access to Education:

In Bhamaragadh, the literacy rate is 46.59%, with female literacy even lower at 19.25% (Government of India, 2011). Older girls frequently care for younger siblings and are married off early due to a lack of education. This educational gap in the tribal community limits knowledge on family planning and modern health, making Madia-Gond children prone to health issues and outdated practices. Table 1 (quotes 5 and 6) describes how children are often kept at home and away from school, especially young girls, who end up participating in household activities.

### 3.2 The Adolescent Experience of Madia-Gond Girls

With multiple influencing factors at socio-cultural and community levels, puberty to late adolescence is a vulnerable time for Madia-Gond girls’ physical and reproductive health.

#### 3.2.1 Socio-Cultural Level

##### Restrictive Cultural Practices during Menstruation Increasing the Risk for Infection:

In Madia-Gond culture, girls and women are isolated in small “Kurumaghar” (Menstrual Huts) during menstruation, which are cramped and can be dangerous, especially in the rainy season due to threats like snake bites. Many women, despite disliking these traditions, are compelled to follow them, affecting their reproductive health, and increasing their vulnerability to infections, as highlighted by the dangers outlined in Table 1 (quotes 7 and 8).

##### Teenage Pregnancies and Early Marriages:

Madia-Gonds have a permissive culture regarding partner selection. Upon puberty, tribe members can freely form premarital relationships. Girls and boys socialize and form relationships, often at community centers during “Rela” cultural events, held 3–4 times a year at “Pandum” festivals. Here, they dance together, and boys’ express interest by offering girls Kharra, a scented tobacco. As shown in Table 1 (quote 9), couples formed during the Rela night often spend the next few days away from the village. Table 1 (quote 10) depicts that in the Madia-Gond culture, a relationship is deemed genuine when a girl becomes pregnant from it. Traditional practices encourage pregnancy in young girls to solidify future relationships, even if this poses danger to the girl. A lack of sexual literacy in tribal adolescents and physical health often results in teenage pregnancies with adverse health outcomes. The World Health Organization (WHO) indicates that teenage pregnancies contribute to higher rates of low-birth-weight babies, still births, miscarriages, and neonatal mortality (WHO, 2023). Madia-Gond tribal girls often begin childbearing in their early teens, with the first pregnancy sometimes occurring by age 15. Marriage age is not fixed, and many have multiple pregnancies before turning 20.

##### Cultural Barriers related to Family Planning, Abortion Restrictions, and Access to Care during Pregnancy:

In the Madia-Gond community, unwed pregnant girls typically choose home births due to fears of mistreatment at healthcare centers. Cultural norms prevent tribal women from considering abortion, viewing it as a sin (Table 1, quote 11). So, family planning methods are not adopted or known, leading women to accept all pregnancies, even if they are dangerous to the mother. Some women even resort to using tablets from informal practitioners or traditional herbs secretly to abort, leading to potential dangers like severe blood loss and infections due to incomplete abortions that could be fatal. Due to a lack of knowledge about postpartum care and home-based neonatal care practices, young teenage mothers are vulnerable to health risks associated with handling pregnancy, delivery, and related complications on their own. As a result, there is a pressing need for better sexual education and healthcare support within the Madia-Gond community to improve the health outcomes of young mothers and their children.

### 3.3 The Adult Experience of Madia-Gond Women

#### 3.3.1 Socio-cultural Level

##### Impact of Traditional Practices on MCH:

In Madia-Gond culture, the consumption of tobacco and alcohol is deeply ingrained in festive and marriage celebrations, and women are not exempt from these practices. Table 1 (quotes 12 and 13) exemplifies the role of tobacco in the lives of Madia-Gond women. Tobacco serves as a means of relaxation during tasks like paddy cultivation firewood transportation, leading In Gadchiroli, 26.5% of women above 15 consume tobacco throughout their lifetime (NFHS-5, 2020). This leads to children being exposed to these harmful substances at a very young age. Prenatal exposure to smoking and alcohol has been linked to sudden infant death syndrome, birth defects, brain damage, and low birth weight (Dodge, 2019). These harmful substances can haveto adverse effects on maternal health, as well as on the health and development of the unborn child. The use of tobacco continues during pregnancy and in the post-delivery period, posing significant risks to both the mother and the baby.

Outdated birthing practices also pose risks to the mother and child. Traditionally, home deliveries in these communities are conducted by untrained birth attendants known as “dais.” A tribal mother describes in Table 1 (quotes 14 and 15) how dais deliver babies using bare hands, cut the umbilical cord with unsterile blades, and use threads or long hairs as cord clamps. The belief in using ash from burnt leaves or dried cow dung for cord healing reinforces age-old practices. Yet, these methods pose post-delivery infection risks to both newborns and mothers, as weak and premature babies are highly vulnerable to infections such as pneumonia, diarrhea, and sepsis (University of California San Francisco Health, 2021). In the community, new mothers receive guidance from older women who have perpetuated traditional practices for generations. These older women firmly believe in the effectiveness of “pujaris” or traditional healers. As shown by Table 1 (quote 16), seeking treatment from pujaris does not involve monetary expenses or the physical and mental stress of navigating multiple health facilities. Due to their low cost and quick response in emergencies, “pujaris” are the preferred choice for seeking treatment for any kind of illness. One tribal member in Table 1 (quote 17) states that pujaris are often utilized simply due to ease of access, not expertise. While some traditional practices prove beneficial, most are not effective. As a result, many tribal people suffer without seeking proper medical care from either pujaris or health facilities. Local health staff hold a negative perception of the community’s preference for healthcare services from pujaris, believing they lack the knowledge and resources to make informed healthcare decisions. Table 1 (quote 18) illustrates the experiences healthcare workers have with tribal members preferring pujaris to allopathic medicine.

#### 3.3.2 Community Level

##### Experiences with Government’s Schemes and Programs:

The implementation of government schemes and programs in tribal areas is challenging because of logistic and operational challenges in remote villages. The Janani Suraksha Yojana (JSY), a cash incentive scheme, is designed to reduce maternal and infant mortalities by encouraging institutional deliveries and care via cash at delivery (Gupta e al., 2012). Table 1 (quotes 19 and 20) shows that tribal women face challenges accessing entitlements even when choosing institutional deliveries. Many also lack bank accounts, making them ineligible for JSY funds.

##### Limited Access to Healthcare Facilities and Transportation Barriers:

Tribal women view managing numerous pregnancies and childrearing as routine. Amid financial strains, cultural barriers, and limited health awareness during pregnancy, self-care often becomes secondary, making tribal women high-risk mothers. Antenatal care (ANC) visits are essential for pregnant women’s safety, diagnosing high-risk conditions like pre-eclampsia and severe anemia. Tribal women underutilize these services, leading to increased health risks. Madia-Gond women struggle with healthcare access due to limited facilities and remote locations. In emergencies, they seek nearby health centers but often find them inaccessible or understaffed. Deliveries typically happen at Primary Healthcare Centers (PHCs) or rural hospitals, which can be up to 40 km away, with distance exacerbating complications. Inefficiency in diagnosing and treating high-risk pregnancies leads tribal women and families down a dangerous path, even resulting in death in the case presented in Table 1 (quote 22). Table 1 (quote 21) explains that the situation worsens during the rainy season when floods block roads and isolate villages, further endangering the chance of survival.

Adequate care and awareness during the antenatal phase significantly improve pregnancy outcomes. In our study, all respondents registered at ANC clinics, but few knew their last menstrual period or estimated delivery date, with only 10% of tribal women fully utilizing ANC services (Dasra, 2016). Women also faced challenges in complying with the minimum four essential ANC visits due to their distance from sub-centers. Difficult terrain and insurgency described in Table 1 (quote 23) prevented health department personnel from reaching pregnant women, leading to no provision of services. The absence of ultrasound services in local health centers compounds access issues. Splitting ANC and sonography appointments requires women to make repeated visits, leading to lost time and wages from household tasks. This deters tribal women from using ANC and sonography services. Table 1 (quote 24 and 25) shows how pregnant tribal women prefer to travel long distances, often by foot, bicycle, or motorcycle, to access better food and health services at NGO hospitals. Table 1 (quote 26 and 27) highlights the transportation challenges and perilous routes to the hospital and back home.

##### Inadequate Nutritional Support by Government Facilities:

The expected nutritional support for the undernourished women at government facilities does not work as efficiently as needed. Women (Table 1, quote 28) noted their negative experience with the government healthcare worker not providing promised food to the tribal people, leaving young mothers in a position where they are unable to provide nutritious meals to their family. Many tribal villages also lack government-supported rural childcare centers. Table 1 (quote 29) shows how tribal women are forced to walk 4–5 kilometers to access minimally nutritious meals of rice, lentils, vegetables, and eggs.

##### Healthcare Providers’ Behaviors and Attitudes:

Community healthcare workers (CHWs) face challenges in promoting health due to the behavior of other healthcare providers. Table 1 (quote 30) captures a CHW’s negative experience with a local tribal doctor, worsened by the poorly maintained facility. The unwelcoming attitudes of health professionals, as shown in Table 1 (quotes 31– 34), hinder women’s access to care. The absence or inefficiency of health staff leads to multiple referrals, wasting time and resources, especially in emergencies. Such experiences demotivate tribal women from seeking public health services.

#### 3.3.3 Interpersonal/Relationships Level

##### Double Burden of Household and Labor Work During Pregnancy:

Pregnant women shoulder a double burden of household work and labor alongside their husbands or families, leading to simple yet difficult lives. Table 1 (quote 35) depicts the strenuous labor that pregnant tribal women endure. This additional workload adds risks to women’s lives when they are pregnant. In this study, a significant number of women are homemakers, yet they carry out strenuous work both at home and in the workplace for little pay. Table 1 (quotes 36–38) describes how women annually partake in farming, fetch water from distant sources, and gather forest products like Tendu leaves. Despite the low earnings, this work is vital for household survival, requiring even pregnant or lactating mothers to contribute. The demanding and grueling lifestyle takes a toll on women’s physical health, although they gradually adapt to the lifestyle over the years. From one account in Table 1 (quote 39), a CHW describes the life-threatening situations Madia-Gond mothers face as they labor through their pregnancy. Such a strenuous schedule significantly increases the likelihood of poor health outcomes, including preterm births, low birth weight babies, and fatalities (Centers for Disease Control and Prevention (CDC), 2023).

##### Access to Inadequate Nutrition During Pregnancy and Post-Partum Period:

Despite their strenuous work, Madia-Gond women’s nutrition is inadequate in quality and quantity. They primarily consume food with little nutrition like rice, diluted lentils, and occasionally wild vegetables and meat. Table 1 (quote 40) highlights how poverty and scarce food availability often mean women eat less. Cultural dietary restrictions during pregnancy further limit their intake, barring certain vegetables, fruits, and meats. Breastfeeding mothers traditionally follow a postpartum diet of soft rice with salt and occasionally red chili powder for days to months (quote 41). The belief is that white rice helps stimulate more breast milk production and is easily digestible. However, this cultural practice contradicts various maternal and child health guidelines, which recommend providing mothers with a nutritious diet in sufficient quantities to aid post-delivery recovery (Quam and Anderson-Villaluz, 2021). Maternal malnutrition significantly impacts the health of newborns, especially low birth weight babies. Ideally, a breastfeeding mother should eat frequently and consume larger portions to support the energy demands of breastfeeding (Quam and Anderson-Villaluz, 2021). However, these women adhere to traditional rules and restrictions passed down through generations in their community. Many women state their shared experience (Table 1, quote 42) in which family members push traditional practices at the expense of the health of themselves and their children. Furthermore, some women even experience such lack of nutrition in the post-partum period, contributing to health issues like anemia in the mother and malnutrition in infants, as explained by Table 1 (quotes 43 and 44).

## 4. Discussion

This study examined factors associated with maternal and child health problems in tribal areas in India. The study utilized qualitative methodologies to analyze the diverse interactions between well established Government healthcare systems and tribal mothers’ and children’s access to care. Applying a life course framework (Jones et al., 2019) as the analytical lens, the study revealed a trajectory of mortality and morbidity risks, from birth to adulthood. The challenges that tribal women and children face are intricately linked to the geographical isolation of tribal villages, the prevailing state of poverty, the dependence on traditional healing practices, insufficient nutrition, and the demanding nature of both domestic and occupational work. The findings emphasize the pressing requirement for holistic interventions aimed at tackling these intricate challenges, with the goal of improving the health and well-being of tribal women and children in India.

### 4.1 Factors Related to Health Risks in Adolescence and Adulthood

Women described, through lived and shared experiences, the trajectory of their health across the different stages of life, commencing from childhood and adolescence. These narratives provided insights into factors that place tribal women at risk for poor health outcomes. For instance, a lack of sexual literacy in tribal adolescents often results in teenage pregnancies with adverse health outcomes. The World Health Organization (WHO) indicates that teenage pregnancies contribute to higher rates of low-birth-weight babies, still births, miscarriages, and neonatal mortality (WHO, 2023). Early marriages and traditional practices encouraging pregnancy in adolescence, placed Madia-Gond girls at risk for adverse pregnancy outcomes. Further, the practices of tobacco and alcohol consumption at a very young age posed a risk to young mothers and their children. In Gadchiroli, 26.5% of women above 15 consume tobacco throughout their lifetime (NFHS-5, 2020). This leads to children being exposed to these harmful substances at a very young age. Prenatal exposure to smoking and alcohol has been linked to sudden infant death syndrome, birth defects, brain damage, and low birth weight (Dodge, 2019). Moreover, the mortality and morbidity risks among the tribes were also driven by the challenges and barriers they encountered in accessing healthcare services. In this study, participants shared that insufficient economic means, impoverished conditions, transportation barriers and an adherence to traditional healthcare practices created obstacles in obtaining optimum maternal and child healthcare services. This is supported by prior research that found that only 10% of tribal women fully utilized necessary ANC services (Dasra, 2016). Additionally, the lack of educational opportunities detrimentally impacted health and well-being of Madia-Gond women, stemming from their restricted knowledge of available healthcare services. This problem is evident in Bhamaragadh, where literacy rates are alarmingly low at 46.59%, and even more so for women, at a mere 19.25% (Government of India, 2011). A similar pattern is observed in Gadchiroli, where a mere 32.7% of women are informed about family planning services, resulting in a substantial deficit in education about safe family planning techniques (NFHS-5, 2020).

Poverty among the tribes also results in insufficient nourishment for women in prenatal and postpartum period. This often presents itself as anemia, a condition suffered by 65 % of tribal women (The Expert Committee on Tribal Health, 2023). The insufficiency of nourishment is further intensified by the obligation for tribal women to engage in labor to sustain the household. The combination of anemia and strenuous labor places added strain on both the expectant mother and the developing fetus (Center for Disease Control and Prevention (CDC), 2023). Further, maternal malnutrition significantly impacts the health of newborns, especially low birth weight babies. Ideally, a breastfeeding mother should eat frequently and consume larger portions to support the energy demands of breastfeeding (Quam and Anderson-Villaluz, 2021). However, these women adhere to traditional rules and restrictions passed down through generations in their community. In a study in North India, maternal mortality rate in the tribal region was primarily attributed to anemia worsened by unhygienic labor practices and incorrect guidance from traditional healers (Chauhan et al., 2012). Traditional healing methods increase the risk of post-delivery infections for both mothers and newborns. Newborns, particularly those who are weak and premature, are highly susceptible to infections like pneumonia, diarrhea, and sepsis (University of California San Francisco Health, 2021). Research undertaken among tribal women in Odisha, India revealed that merely 6% of the surveyed tribals solely opted for allopathic medical treatments (Mahapatro et al., 2000). Instead, 49% of the participants leaned towards traditional remedies, often administered by local unlicensed practitioners (Mahapatro et al., 2000). Adherence to traditional remedies that lack scientific evidence increases mortality and morbidity risks. A study conducted in Bastar, Chhattisgarh, a locale where 70% of the populace comprises tribal communities, revealed a stark contrast in maternal mortality rates. Among tribal women, the maternal mortality rate stood at 100%, while among non-tribal women, it was reported to be 0% (Chauhan et al., 2012). Further, geographical isolation, insufficient transportation framework, and great distances to healthcare establishments present noteworthy difficulties for tribal populations in their pursuit of maternal and child healthcare services.

### 4.2 Factors Related to Health Risks in Childhood

The glaring disparities in child health outcomes among tribal populations, particularly concerning child mortality rates, are associated with factors such as traditional childcare practices, limited access to nutritional diet and healthcare services and limited access to education. The Infant Mortality Rate (IMR) among tribal children surpasses the national average of India by 30% (Dasra, 2016). Bhamaragadh’s neonatal mortality is high, accounting for 12.4% of Maharashtra’s total neonatal deaths (Saunik et al., 2017). Furthermore, a child born into a tribal family has a 19% higher chance of dying in the neonatal period and 45% higher risk of dying in the post-neonatal period compared with children of other social groups (Narain, 2019). These alarming statistics underscore the critical need to address the underlying factors contributing to such disparities and to devise strategies that specifically cater to the healthcare needs of tribal children. Madia-Gond children, as well as their counterparts from other tribal communities, confront health challenges marked by inadequate nutrition, substandard living conditions, heightened vulnerability to infectious diseases and complications from outdated traditional practices. The scarcity of essential nutrients, often attributed to economic constraints and limited access to nutritious food sources, contributes to stunted growth and compromised immune systems among these children. For instance, in a study in India, a significant percentage of tribal children, approximately 53%, suffered stunted growth, while 29% experienced severe stunting, and 55% were underweight (Nikitin et al., 2010). Moreover, only 4.5% of tribal children aged 6–23 months receive an adequate diet, manifesting in 35.4% of children being underweight (National Family Healthy Survey-5 [NFHS-5], 2020). The lack of nutritious food also causes developmental delays and impairments in cognitive functions, with the under-5 stunting rate in the rural Gadchiroli district being 35.7% (NFHS-5, 2020). This nutritional deficiency is exacerbated by substandard living conditions, characterized by overcrowded and unsanitary environments, which create a conducive breeding ground for infectious diseases. Furthermore, the persistence of outdated traditional practices, rooted in tribal cultures, expose these children to preventable health risks. Practices such as unhygienic birthing methods and reliance on traditional healers can lead to complications during childbirth and untreated infections, heightening the vulnerability of tribal children to serious health issues (Kumar et al., 2020). Addressing these interrelated challenges requires comprehensive interventions that encompass improved nutritional support, enhanced living conditions, and culturally sensitive healthcare practices tailored to the unique needs of tribal communities and their children.

### 4.3 Access to Healthcare Services

To foster change and improve maternal and child health outcomes in Bhamaragadh and other tribal regions, a multi-faceted approach is required. Policy interventions should focus on improving healthcare infrastructure and services in the region. There is need for comprehensive maternal care initiatives, encompassing strengthened antenatal and postnatal care and nutritional guidance and access among the tribes (Kumar et al., 2020). Increasing the number of trained healthcare professionals, including skilled birth attendants, and strengthening referral systems to provide timely and quality emergency obstetric care can significantly reduce maternal mortality rates among tribal populations. Redesigning parts of the public healthcare sector to include monetary and nonmonetary incentives can encourage healthcare workers and physicians to work in remote tribal areas (Kumar et al., 2020).

Nutrition interventions are also crucial to combat malnutrition in these tribal communities. The implementation of government schemes such as the Integrated Child Development Services can play a pivotal role in providing nutritious food, immunization, and growth monitoring for children (Sachdev & Dasgupta, 2001). Additionally, promoting breastfeeding, educating mothers about appropriate childbearing practices, and encouraging family planning are vital steps in preventing maternal and child mortality (Agampodi et al., 2021). In essence, conducting extensive research on the healthcare disparities experienced by tribal communities holds the potential to wield significant influence on policy formulation and funding allocation. Such research insights can pave the way for targeted improvements in healthcare infrastructure, thereby facilitating increased accessibility to essential medical personnel, nutritious sustenance, and educational resources in remote tribal-populated regions.

### 4.4 Strengths and Limitations

The strength of this study lies in its utilization of a wide range of engagements with Madia-Gond tribal women spanning different age groups, as well as individuals from diverse professional backgrounds and levels of organizational engagement. This approach offers a comprehensive and varied perspective on maternal and child-health related issues and barriers to access to care. The limitations encompass the restriction of data collection to a district in the state of Maharashtra. Given that the tribal population constitutes 8.6% of India’s population, differences in geography and culture could potentially contribute to distinct challenges in each tribe (Ministry of Tribal Affairs, 2023). However, 40.6% of tribal communities reside below the poverty line, while 90% of these populations inhabit remote rural regions, presenting similar obstacles to those encountered by the Madia and Gond people in terms of healthcare accessibility (The Expert Committee on Tribal Health, 2023). Efforts are needed to increase healthcare infrastructure, funding, and education targeting the health and well-being of tribal women and children. An additional limitation pertains to the exclusion of data directly obtained from minors in the study. Instead, this investigation relied upon data sourced from young adults and mothers, capturing shared experiences spanning their infancy and childhood, along with insights gained from their roles as mothers to young children. Despite the limitations, this study presents a distinctive perspective valuable to researchers, practitioners, and policymakers in their pursuit of solutions for maternal and child mortality issues within tribal communities. This research introduces innovation through its application of a life-course methodology, delving into the influence of tribal individuals’ distinct lifestyles and customs on maternal and child health outcomes, while also pinpointing the barriers obstructing women’s healthcare access.

## Figures and Tables

**Figure 1.**
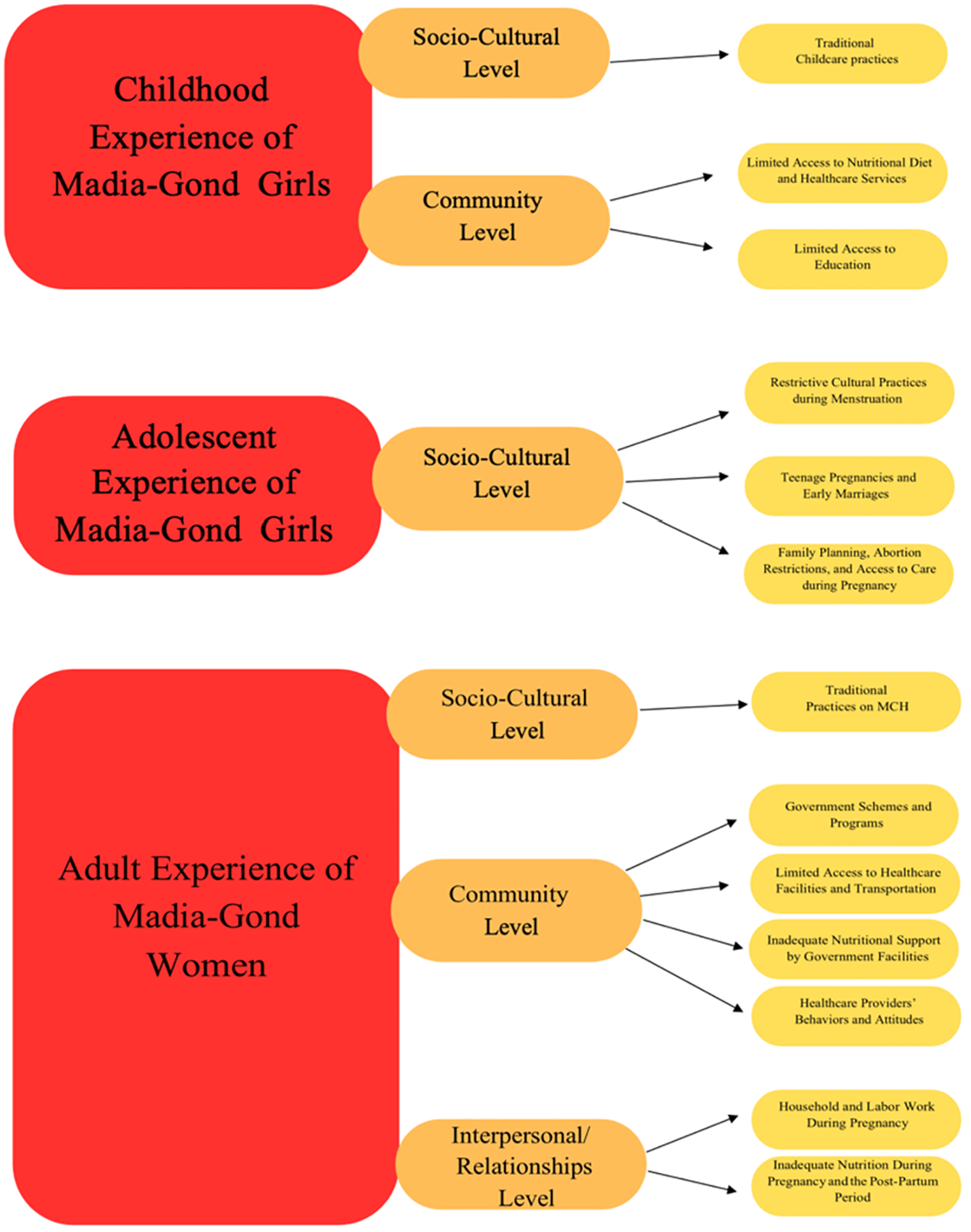
Health Risks for Madia- Gond Women and Children

## Data Availability

The data that support the findings of this study are available on request.

## Appendix

**Table 1.** Example Quotes from participants

Life-Course Phase	Ecological Level	Theme	Respondent Characteristics	Quote Number	Example Quotes
Childhood phase	Socio-Cultural Level	Impact of Traditional Childcare Practices	Tribal Mother from Marampalli; Age 23	1	*“My mother asked to extract out the foremilk and the thick yellowish milk immediately before breast feeding the baby as it is considered not good milk for a newborn. It may not be palatable or it may get stuck in the mouth due to its stickiness.”*
			Tribal Mother of a Deceased Baby Boy from Pengunda; Age 23	2	*“I delivered a baby boy at home, local Dai (midwife) performed the delivery. Until the umbilical cord falls of the naval, the baby and mother are kept separately isolated in a dark room away from the sight of other family members. It is a usual part of our culture. The very next day my baby boy started crying non-stop. I could not understand what had happen to him. That whole day my baby cried and around 11 pm in the night he died.”*
	Community Level	Limited Access to Nutritional Diet and Healthcare Services	Tribal Mother from Kumarguda; Age 27	3	*“Most days, both my children have a small bowl of rice and very little sabji (vegetable) or dal (pulses).”*
			Tribal Mother from Kothi; Age 19	4	*“Yes, we buy dal (pulses) of around INR 40 in a week. It’s very unaffordable to us and cannot buy more than that. Yes, we produce rice and that too only during rainy season. From where do we get water for farming in other seasons, when we do not even have water to drink.”*
		Limited Access to Education	Tribal Mother from Middapalli; Age 33	5	*“I never went to school. Since childhood I used to help my mother with household chores, fetch water, Sarawane (mopping the floor with cow dung liquid), and go to the forest with my mother for the collection of chaar (the Cuddapah almond). Actually, none of my friends attended any school ever, as it was very far from our village with no proper approach road. So we all used to help our mothers in household work.”*
			Key Informant from Laheri; Age 26	6	*“In their culture mostly, older girls are busy looking after their siblings. These girls do not get exposed to the outside world, they are very shy and scared to mingle with outsiders. Almost all of them do not attend school. They are of great help to their mothers right from their early age. It is because of them their mothers can go for agriculture labor work by keeping their younger ones in custody of the elder daughters.”*
Adolescence phase	Socio-Cultural Level	Social Isolation During Menstruation Increasing the Risk for Infection	Young Woman from Juvi; Age 25	7	*“Last year one teenage girl died of snakebite during rainy season while asleep in Kurumaghar, immediately she screamed that she was bitten by a snake but her father could neither touched her nor take her to hospital as she was menstruating at that time. Touching a menstruating woman is bad sin in our tradition. This is why we don’t like sleeping separately in Kurumaghar. We feel sad and depressed during our monthly menstrual cycle as we are treated differently and kept in isolation for the days of menstruation.”*
			Tribal Mother from Bhamaragadh; Age not available	8	*“In our village we need to get up very early in the morning during monthly periods, need to clean ourselves in the river before sunrise to avoid men’s sight. We really hate ourselves during our monthly cycle.”*
		Teenage Pregnancies and Early Marriages	Key informant from Maraknar; Age 52	9	*“The young girls and boys go for”Rela”. Rela is amusement nights on specific seasons for youngsters to select their life partners. The youngsters are free from having any consent of their parents for attending the ‘Rela.’ During this gathering they select their partners and even can perform sex as requisite for getting intimate to each other or vice versa. After this incident if a girl gets pregnant, their relationship is considered serious. So, they either get married or the girl can go to a boy’s home without the marriage. They can continue to stay together as husband and wife without any inhibitions of society.”*
			Key informant from Malampoddur; Age 45	10	*“If a young girl gets pregnant early in a relationship, only then it is considered as a sign of their deep love and strong relationship between the boy and girl. The girl chooses the boy as her life partner.”*
		Cultural Barriers Related to Family Planning, Abortion Restrictions and Access to Care During Pregnancy	Tribal Mother from Kosfundi; Age 28	11	*“Once we get pregnant, we must keep the pregnancy. Culturally we are not allowed to abort the pregnancy, it is considered as sin. Baby is a god’s gift; we should not go against god’s will.”*
Adulthood phase	Socio-Cultural Level	Impact of Traditional Practices on Maternal and Child Health	Key informant from Botanfundi; Age 27	12	*“They consume tobacco because there is high illiteracy among them and high peer pressure for tobacco consumption…. They even commonly consume tobacco during their pregnancy.”*
			Tribal Mother from Kothi; Age not available	13	*“I have consumed it since my childhood but just a pinch of it. At least a pinch is needed in an hour or two. We friends used to go into the jungle for firewood collection and in between we often used to have the tobacco as time-pass. That’s how I am consuming tobacco. It gives me some relief from strain or pain during our work.”*
			Tribal Mother from Nargunda; Age not available	14	*“Dai performed the delivery… cut the cord with a new blade and tied it with white thread. My mother-in-law burnt some dried leaves in the fire of the chulha (fire stove), collected its ash and put it on the cut portion of the cord.”*
			Tribal Mother from Tirkameta; Age not available	15	*“I delivered at home; my mother-in-law helped me deliver the baby. She tightly tied the cord with a few of my long hairs and just cleaned the baby with wet cloth.”*
			Tribal Member from Pidmili; Age not available	16	*“If we intend to go to a nearby health facility there is only one bus in a day, if we miss it, we have no other option except a bike. There are hardly 3–4 households in the village that owns bikes. Moreso, we are unsure if we could get any treatment in the sub center. We often find that ANM tai is not available in sub centers, So we lose both our money and time. Hence, we prefer going to Pujari, he does not charge us money and moreover he stays in the village only. We only have to carry some offerings to our deity.”*
			Tribal Member from Pidmili; Age not available	17	*“The nearest health center is very far; we have no transport facilities available here in the village. Also, if we get late because of treatment in an NGO hospital then we resort to stay back at night in the hospital itself, so it’s better to visit our village pujari.”*
			Key informant from Nelgunda; Age 45	18	*“Sometimes women listen to us and sometimes don’t. Their family members do not pay that much attention. Pujaris are given more importance than us.”*
	Community Level	Government Schemes and Programs	Tribal Mother from Nargunda; Age not available	19	*“It’s been over six months now but I did not receive any money in my account from the scheme for delivered women. My husband is constantly checking our bank account at Bhamaragadh. He went a couple of times to Bhamaragadh to check the money in our account but there wasn’t any, instead we spent good money on the travel.”*
			Key informant from Mannerajaram; Age 35	20	*“They have no other means of livelihood but to earn money from daily wages. They have been issued a worker’s card (Scheme MGNREGA card) but there seems no work there in the village or nearby places. so they tend to migrate in search of employment when there is no agricultural work for them. The MGNREGA is to supposedly provide 100 days (@ INR 274 / day) work in a year.”*
		Healthcare Facilities and Transportation	Tribal Mother from Nelgunda; Age 18	21	*“Our village has no proper road and is located in the deep forest. We must walk a long distance to go to the hospital. In the rainy season Juvi water canal gets flooded…no one can pass through that. For very critical cases our men put women on a khat (wooden framed cot) and wade through the water only if water level is below the chest. The last resort is to take it to either pujari or the Dai or leave it to her fate either service or rest in peace.”*
			Tribal Family Member from Gerra; Age not available	22	*“The lady was crying in severe pain throughout her journey in the ambulance to the Aheri rural hospital. I and one nurse were sitting beside her. At Aheri hospital, she delivered a still baby and slipped into a critical condition. As an emergency the doctor further referred her to another facility at a distance of an hour, we reached the hospital, and staff took her to an ICU bed where she breath last.”*
			Tribal Woman from Kawande; Age 19 years	23	*“We live in a very interior village where there is no proper road connectivity or transport facility. The nearby sub-center opens sometimes in a month. We have no other health facility nearby to go for our health check-ups.”*
			Tribal Mother from Tirkameta; Age 21	24	*“There is no proper public facility available nearby and the one which is there is in very poor condition with its rooms and beds. The staff do not behave well with us, we get offended and avoid going to this facility, we prefer to go to the NGO hospital which is far away.”*
			Tribal Mother from Arewada; Age not available	25	*“We like to deliver in ‘Lok Biradari’ (NGO hospital) as they take good care of us and provide full meals after delivery. Also, nurses are good but sometimes in emergency situations, we deliver at home. We have no option.”*
			Tribal Mother from Visamundi; Age 26	26	*“We borrowed a bike from our relatives in the village, filled the petrol ourselves and reached the NGO hospital for delivery. But most of the time it is not possible if the bike is not available, last time I delivered at home without giving much trouble to the family members. We do not have a network to our cell phones in our village. It’s difficult to even call an ambulance. Things get worsened due to poor roads to our village and the villagers are left on to use the narrow trails through dense forest.”*
			Tribal Mother from Dhudepalli; Age not available	27	*“They got me into the tractor, as an ambulance was not available at that time. Somehow, we reached the Primary health center. But they referred us to the NGO hospital. After delivery, we traveled (8 km) back by bus and then we walked with newborn baby holding in hand through forests on a tinny rough road for about two hours (over 10 kms) to our hamlet.”*
Adulthood	Community Level	Nutritional Support by ICDS centers, Government Facility	Tribal mother from Pengunda; Age not available	28	*“Our village Anganwadi worker [community health worker]does not properly provide food to us and our kids, she provides just rice and dal. But It’s been a year now and we have not gotten anything from Anganwadi.”*
			Tribal Mother from Botanfundi; Age 28	29	*“I learned they cook food like rice and sabji [vegies] in Anganwadi, but it is too far from my home, I have three small kids and no one to take care of them at home, that is why I do not go to the center to avail that food.”*
		Healthcare Providers’ Behaviors and Attitudes	Key informant from Mannerajaram; Age not available	30	*“The main doctor is never present in the primary health center (PHC), he hardly visits here. He behaves very rudely and scolds us many times. Also, other doctors give only one or two medicines for all types of illnesses. Often, they do not have medicine stock available with them. In PHC there is no electricity, so the doctor keeps the vaccines in the refrigerator of people known to him in the surrounding area as the PHC does not have an ILR.”*
			Tribal health Worker from Dhudepalli; Age not available	31	*“Whenever we go to the PHC no staff is available there. Often the peon has to go and call them from their respective homes if any patient comes to the health facility, we have to wait for hours and get insulted upfront. They do not treat us well. We only come to visit the facility in an emergency situation, what else can we do.”*
			Tribal family member from Brahmanpalli; Age 22	32	*“We do not have any other health facility nearby except PHC, but the PHC is also ill equipped with necessary things and the main doctor is almost unavailable there. Their services are so poor that we don’t wish to visit the PHC at all. The support staff is also rude. But still, we go to the PHC only. We are poor people, where else could we go?”*
			Key informant (location unavailable); Age not available	33	*“Yes, Bengoli doctor (Quack) does visit our homes in case of emergencies and otherwise also, although he charges high, but he keeps money on credit as well.”*
			Key informant from Tirkameta; Age 40	34	*“Government ANM does not come to visit pregnant girls at home even once throughout pregnancy…she asks me to call these pregnant girls/women at Anganwadi center, which is 5 km away from their village. What skills do I have compared to the ANM, I just come and monitor if these girls are okay or not.”*
	Interpersonal/Relationships Level	Household and Labor Work During Pregnancy	Key informant from Botanfundi; Age 27	35	*“We do home visit and advise pregnant women to take at least 2 hours rest in a day, eat 3 to 4 times a day, but they do not take care of themselves, they remain so busy throughout the day in household work. Also, many can’t afford to have food 3–4 times a day even if they feel hungry. These tribal women are so patient and silent sufferers.”*
			Key informant (location unavailable); Age 32	36	*“The women in the community work like hell, they do not even care during pregnancy. They even go for Cheeronji (Cuddapah almond) and Tendu patta (Indian Ebony) collections in the third trimester of pregnancy. We always tell them to take rest for at least 2 hours but very few women follow that.they are feared of losing their wages.”*
			Tribal family husband from Botanfundi; Age 30	37	*“During peak season almost everyone in the village goes for Tendu patta (Indian Ebony leaves). Tendu patta contractors pay us well and they are the only source for short duration but with good income in a year. Thus, the whole family including pregnant women and postnatal mothers and even children above 5 years old goes for Tendu patta collection.”*
			Key informant from Kothi; Age 32	38	*“It’s the woman in the house who works day and night, takes care of the family, and supports family income. Men are busy hunting, fishing, drinking Mahua liquor, and also beating their women. Women in the tribal community are busy with household chores right from firewood collection, fetching water, animal husbandry, cooking etc. which continue throughout pregnancy and till the day of delivery until they get into labour. Some such situations put them in emergency conditions and they end up delivering in the field of the jungle*.”
			Key informant from an NGO; Age 25	39	*“This is her 8th term to deliver; she has become very weak. Despite that, she must work very hard throughout the day. Her husband drinks a lot, and they are very poor. We advised her not to take any one chance, but she did not go for a family planning operation.”*
		Nutrition During Pregnancy and Post-Partum Period	Key informant from an NGO; Age 25	40	*“We eat thrice, only if the food is available otherwise, we eat twice a day, and mostly have Ambil (grain liquid like porridge) in between but eventually Ambil is available only in summers. Yes, during pregnancy also.”*
			Tribal Mother from Mannerajaram; Age not available	41	*“It has been one week now; they give me very little rice and that too soft rice mixed with chili chutney. I do not like it, but I had to eat it, they do not give me other food.”*
			Tribal Mother from Jinjgaon; Age 23	42	*“My mother-in-law does not allow me to eat a full meal at a time, she gives me soft rice mixed with salt in a small bowl three times a day, but I was needing more, and I feel hungry at times. She says “do not eat more until a few days after delivery. It’s not good for the baby’s health. Overeating would put pressure on the child and its growth will affect.”*
			Tribal Mother-in-Law from Kothi; Age not available	43	*“She has been eating soft rice with salt and some dry chili powder for around one month. She needs to eat that for a good amount of breast milk secretion in the postpartum period, it is good for the baby too.”*
			Key informant from Botanfundi; Age 27	44	*“They eat mostly rice and vegetables, whatever is available and sometimes dal. They feed only rice to the infants above 7 months. Girls and women are very anemic and weak.”*
